# Pseudonymization tools for medical research: a systematic review

**DOI:** 10.1186/s12911-025-02958-0

**Published:** 2025-03-12

**Authors:** Hammam Abu Attieh, Armin Müller, Felix Nikolaus Wirth, Fabian Prasser

**Affiliations:** Berlin Institute of Health at Charité – Universitätsmedizin Berlin, Medical Informatics Group, Charitéplatz 1, 10117 Berlin, Germany

**Keywords:** Biomedical research, Data protection, Pseudonymization, Software, Tool, Service, Review

## Abstract

**Background:**

Pseudonymization is an important technique for the secure and compliant use of medical data in research. At its core, pseudonymization is a process in which directly identifying information is separated from medical research data. Due to its importance, a wide range of pseudonymization tools and services have been developed, and researchers face the challenge of selecting an appropriate tool for their research projects. This review aims to address this challenge by systematically comparing existing tools.

**Methods:**

A systematic review was performed and is reported according to the Preferred Reporting Items for Systematic Reviews and Meta-Analyses (PRISMA) guidelines where applicable. The search covered PubMed and Web of Science to identify pseudonymization tools documented in the scientific literature. The tools were assessed based on predefined criteria across four key dimensions that describe researchers’ requirements: (1) single-center vs. multi-center use, (2) short-term vs. long-term projects, (3) small data vs. big data processing, and (4) integration vs. standalone functionality.

**Results:**

From an initial pool of 1,052 papers, 92 were selected for detailed full-text review after the title and abstract screening. This led to the identification of 20 pseudonymization tools, of which 10 met our inclusion criteria and were assessed. The results show that there are differences between the tools that make them more or less suited for research projects differing in regards to the dimensions described above, enabling us to provide targeted recommendations.

**Conclusions:**

The landscape of existing pseudonymization tools is heterogeneous, and researchers need to carefully select the appropriate solutions for their research projects. Our findings highlight two Software-as-a-Service-based solutions that enable centralized use without local infrastructure, one tool for retrospective pseudonymization of existing databases, two tools suitable for local deployment in smaller, short-term projects, and two tools well-suited for local deployment in large, multi-center studies.

**Supplementary information:**

The online version contains supplementary material available at 10.1186/s12911-025-02958-0.

## Introduction

### Background

The development of modern data-driven methods in medicine, particularly artificial intelligence (AI), requires access to large datasets [1]. At the same time, the studies that are needed to evaluate the resulting personalized medicine approaches are also becoming more complex in terms of the number of sites and types of data involved [2]. This implies new challenges in terms of both site-specific and cross-site secure identity management. Medical data is sensitive, and its processing is governed by the law [3, 4]. The regulatory framework differs by jurisdiction. In some regions, such as the United States, medical data is explicitly regulated under specific laws like the Health Insurance Portability and Accountability Act (HIPAA) [5]. In contrast, in the European Union, medical data falls under the broader scope of personal information as defined by the General Data Protection Regulation (GDPR) [6]. To address these challenges, methods are needed that protect privacy while collecting and managing such data [7].

Pseudonymization is a common technique implemented in biomedical research at the data level. Different laws, regulations, and guidelines recommend or mandate it as a primary data protection mechanism [6, 8]. In contrast to other privacy-enhancing technologies, such as differential privacy, pseudonymization is a privacy-by-design measure usually applied during stages of data collection and integration. Terms such as “pseudo-anonymization” or “linked-anonymization” are used synonymously in some regions. In the context of our work, we follow the definition described in ISO/IEC 20889:2018, which emphasizes the replacement of directly identifying information while maintaining the possibility of re-identification under certain conditions [9]. However, the term pseudonymization can also be interpreted in different ways, such as referring to the removal of directly identifying information without necessarily maintaining a link. In practical terms, pseudonymization means the separate storage of directly identifying data, such as names or personal identifiers, from the data that is required for conducting the scientific analyses [10]. The goal is to ensure that data can be processed in a way that prevents direct attribution to specific individuals when no additional information is available. The identifying information is instead replaced with a unique pseudonym, while the link between the pseudonym and the identifying data is stored securely. This enables the linkage of different data types for the same patients or study participants across different sources and time points [11]. It also allows the re-identification of subjects when necessary and legally permitted, such as in the case of subsequent data collection or re-contacting due to incidental findings [12].

**Fig. 1:**
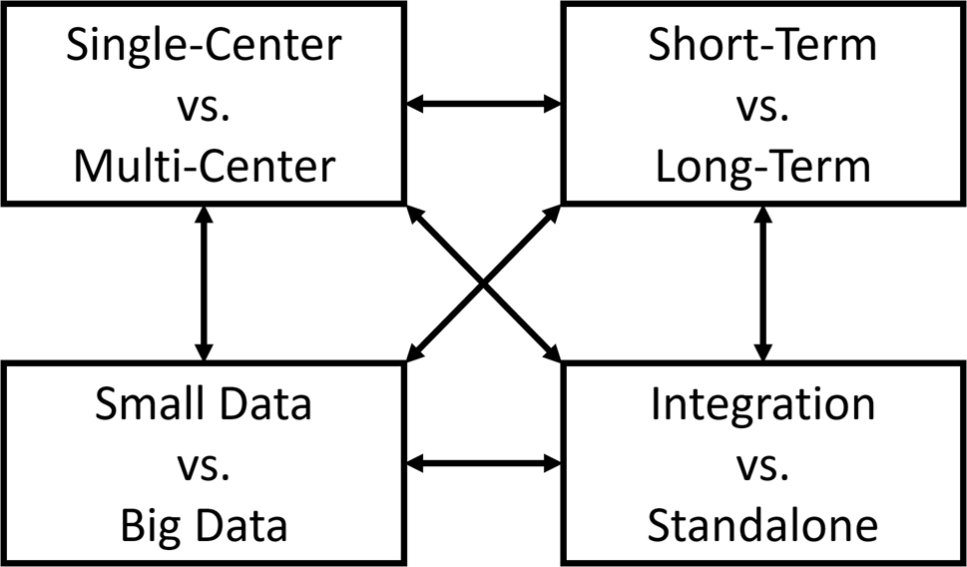
Dimensions for selecting a suitable pseudonymization tool

Choosing the right pseudonymization tool requires careful consideration of various specific aspects. For short-term studies and projects, immediate availability is important. For long-term undertakings, such as the establishment of a sustainable research platform, seamless integration and scalability are essential. In settings focusing on the re-use of routine data, the number of identities that need to be processed can be very high. Overall, these aspects can be categorized into four key dimensions, which are presented in Fig. 1.

The first dimension, (1) single-center vs. multi-center, assesses whether a research activity takes place at a single or spans multiple sites. This results in different needs for pseudonymization, e.g., linkage of data across sites. The second dimension, (2) short-term vs. long-term, refers to the duration of use. Short-term projects focus on quick deployment, such as during health crises like COVID-19, and often include cross-sectional studies or pilot studies with a duration of several months. Long-term projects usually have higher requirements in regards to software maintainability, e.g., through comprehensive rights and roles models and interfaces to long-term storage, and can include longitudinal cohort studies or registry-based research that spans multiple years. The third dimension, (3) small data vs. big data, concerns the volume of the managed data. Projects that process large numbers of existing identities and data records have higher scalability requirements. For example, qualitative interview studies typically fall into the category of small data, while population cohorts with deep phenotyping using images and genomics could be considered examples of big data projects. The fourth dimension, (4) integration vs. standalone, focuses on integration into existing workflows or software. Standalone systems suit smaller projects with minimal external data interaction, while other solutions are better suited for integration into more comprehensive platforms. As indicated by the arrows in Fig. 1, these dimensions are not independent but are often tied to each other, which means that certain project types tend to have common properties that can influence each other. For example, tools designed for big data can typically also handle small data projects, and some tools may be suitable for both short- and long-term projects depending on their configuration and adaptation capabilities.

### Objective

The main objective of this study is to help researchers with finding a pseudonymization tool fitting their specific needs. More specifically, we (1) collected pseudonymization tools that have been described in the literature and are openly available, (2) categorized their technical properties and development status, (3) assessed them regarding their suitability for projects with different requirements according to the dimensions outlined above, and (4) developed recommendations for which tool might be most suitable for which type of project.

## Methods

We performed a structured review and report our results following the Preferred Reporting Items for Systematic Reviews and Meta-Analyses (PRISMA) guidelines wherever applicable [13]. Following a structured search and selection process, we assessed the identified pseudonymization tools based on different properties. We then compared the tools to make recommendations.

### Selection process

As the topic of our review is placed at the intersection of two fields, information technology and medical research, we searched PubMed and the Web of Science Core Collection. Our search string combined the context of pseudonymization with our aim to identify software tools or services. To identify relevant papers, we used two sets of keywords:

(A) Set 1: “pseudonyms” or “pseudonym” or “pseudo-anonymous” or “pseudo-anonymization” or “pseudo-anonymisation” or “linked-anonymous” or “linked-anonymization” or “linked-anonymisation” or “pseudonymity” or “pseudonymization” or “pseudonymisation” or “pseudonymized” or “pseudonymised” or “pseudonymous”.
(B) Set 2: “service” or “tool” or “software” or “application”.

We combined them using AND logic, meaning that at least one term from Set A AND at least one term from Set B had to appear in the title or abstract. The specific search queries for both databases are provided in the Supplementary File 1.

**Fig. 2:**
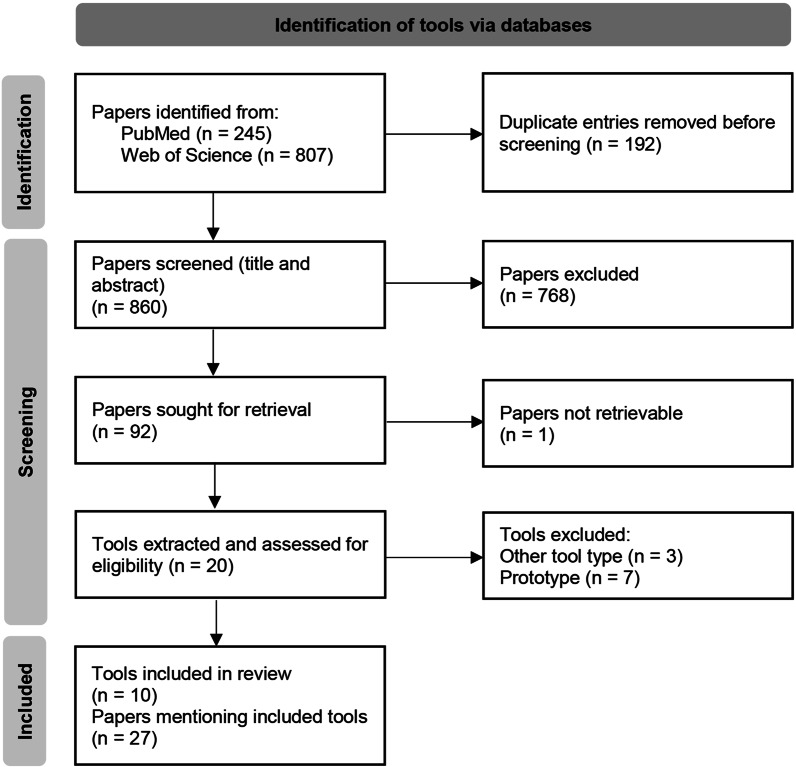
Flow diagram of the selection process for pseudonymization tools (based on [13])

Figure 2 shows the whole screening and selection process, which was limited to peer-reviewed original articles, reviews and overviews in English. The final search was conducted on June, 18^th^ 2024 and resulted in 1,052 articles. The results were exported as comma-separated value (CSV) files and imported into Rayyan, an online tool for collaborative systematic literature reviews [14]. Two sets of inclusion and exclusion criteria were defined – one for the screening of papers and one for the selection of tools.

In a first step, we removed all 192 duplicates. In the next step, we performed a title-and-abstract screening based on the predefined criteria for paper selection. We included articles that

- were original research articles, review articles, or systematic reviews,
- described, mentioned or referenced pseudonymization tools in a medical context, whether through use, development, implementation, evaluation, or discussion,
- were published in English.

We excluded articles that

- were opinion pieces, editorials, or commentaries,
- were published in languages other than English.

In terms of the publication date, there were no restrictions. As a result, 768 articles were excluded. Most excluded papers focused on pseudonymization in other contexts, including vehicle communication technologies (e.g., authentication in vehicular networks) and blockchains, or did not explicitly mention or reference a specific pseudonymization tool. Each paper was screened by two of the authors. In case of uncertainty or disagreement, a third reviewer was consulted to resolve discrepancies and reach a consensus. The third step involved a full-text screening. A formal quality assessment of the selected papers was not performed, as the primary use of the papers was simply to identify pseudonymization tools. From the remaining 92 articles, we extracted 20 pseudonymization tools. These tools were then evaluated based on our predefined criteria for tool selection. We included tools that

- had a primary focus on medical applications,
- were fully developed solutions rather than prototypes,
- primarily focused on registering structured identifying data and generating pseudonyms,
- were publicly available or sufficiently documented to assess their core functionalities.

We excluded tools that

- were designed to automatically identify or remove identifying data, such as Natural Language Processing (NLP) approaches for text documents or image data,
- were plugins, add-ons, or implemented pseudonymization functionalities embedded within broader software solutions.

Most of the tools were excluded as prototypes, NLP-based de-identification approaches or embedded functions in comprehensive software solutions. Ultimately, we included ten tools, which were referenced in 27 papers.

### Data charting

The data charting process was based on the four dimensions described in the background section. To categorize the tools along these dimensions, we selected data items that provide relevant indicators. These include technical data such as details about the pseudonymization algorithm or interfaces as well as meta-data providing contextual information.

Primarily, we extracted the information about the tools from the respective papers. If necessary, a separate internet search was conducted to gather further data. The data extraction was performed by one reviewer and subsequently verified by a second reviewer. An overview of the data items is shown in Table 1. The complete table of all collected data items is available in Supplementary File 2.

**Table 1:** Information items gathered for the tools with their respective definition and examples

Data item	Definition	Examples
***Technical data***		
Support for pseudonym spaces	Ability to generate duplicate-free pseudonyms across different locations or institutional sites.	Yes, No
Support for record linkage	Ability to identify, combine, match, or link records from one or more databases or sources.	Yes, No
Support for secondary pseudonymization	Ability to pseudonymize already pseudonymized data to further enhance privacy.	Yes, No
Support for updates	Ability to update the data managed by the service.	Yes, No
Support for batch processing	Ability to pseudonymize multiple identities with one operation.	Yes, No
Pseudonymization algorithm	Specific method or technique used to generate pseudonyms.	Hashing, Encryption, Random identifiers, Autoincrement
GUI (Graphical User Interface)	Existence and nature of a graphical user interface.	Web-based, Native, No
API (Application Programming Interface)	Existence and protocols for software interaction and integration.	RESTful API, SOAP, No
***Meta data***		
Institution	Institution affiliated with the primary author of the software.	University of Nottingham
Country	Country where the institution associated with the primary author of the software is based in.	Germany, United Kingdom
License	Type of license under which the software is released.	AGPL-3.0, MIT License, GPL-3.0
Release date	Year the tool was released (order of priority: paper publication, source code, other information).	2013
Latest update	Most recent year when the software received an update or upgrade (order of priority: source code, other information, paper publication).	2022

### Tool classification

For the purpose of this study, we distinguished between two general types of medical research projects based on the four key dimensions:

A. Large, complex projects, characterized by multi-center collaboration, medium- to long-term duration, big data processing, and heterogeneous technical infrastructures.
B. Small projects, typically single-center, with short running times, and smaller datasets.

We associated the values collected for the data items with the properties of the four dimensions to identify whether tools are tendentially better suited for projects with the according property. We then used the associations to assign them to one of the two general types of projects. However, this does not imply that their applicability is limited to these project types – many tools can be used in broader contexts. For the assignment, we applied a quantitative scoring method. Each tool received a binary score for each relevant property across relevant dimensions (e.g., 0 = not supported,1 = supported). A total score was then calculated to assess tool suitability. The detailed scoring process is provided in Supplementary File 3.

## Results

### Overview

In this section, we provide an overview of the identified tools in ascending alphabetical order, followed by an assessment in terms of the four dimensions.

#### ALIIAS

ALIIAS, which stands for “Anonymization/Pseudonymization with LimeSurvey integration and II-factor Authentication for Scientific research”, was introduced in 2023 [15]. It provides a web-based interface for pseudonymization and anonymization, which can be integrated with the survey web application LimeSurvey [16]. Installing the software may require familiarity with this platform and advanced IT knowledge, particularly for configuring security mechanisms. The code, last updated in 2022, along with an extensive user manual are available on GitHub [17].

#### CRATE

Clinical Records Anonymisation and Text Extraction (CRATE) was introduced in 2017 [18]. It can be used to retrospectively pseudonymize existing structured data in relational databases using cryptographic methods, with the option to integrate external natural language processing (NLP) tools for free-text data processing. Installation requires database setup, which may pose a challenge for non-technical users. The code, with its latest update in 2024, is accessible on GitHub [19].

#### EUPID

The European Unified Patient Identity Management (EUPID) tool was initially developed by the Austrian Institute of Technology (AIT) and is managed by the European Commission – Joint Research Centre (EC-JRC) [20]. It was recommended by the European Rare Disease Registry Infrastructure (ERDRI) for rare disease research [21]. EUPID is provided as a Software-as-a-Service (SaaS) and can be used in two ways. Users can access the web-based interface on the ERDRI platform, which allows to use tool directly in the browser, making it particularly accessible for non-technical users. Alternatively, it is possible to integrate it into other systems via an API, which may require programming knowledge. The source code is not publicly available, so potential updates cannot be verified. Documentation and workshop materials are accessible on different platforms [22].

#### gPAS – generic Pseudonym Administration Service

Introduced in 2015 by Bialke et al. at the University of Greifswald, the generic Pseudonym Administration Service (gPAS) is a web-based application and service for creating and managing pseudonyms [23]. It supports customized pseudonyms through prefixes, suffixes, various alphabet options, and domains which serve as a semantic grouping of pseudonyms. While gPAS is available as a Docker container, its deployment involves setting up and hosting a server, as well as manual configuration, which requires IT expertise to ensure proper integration into existing infrastructures [24]. The code, updated in 2024, is publicly available on GitHub [25].

#### Mainzelliste

The Mainzelliste, developed in 2015 as a successor to the PID Generator (see below), uses a combination of error-correcting codes, cryptography, and random values to generate pseudonyms [26]. It is also available as a Docker container and requires the setup and hosting of a server as well as manual configuration, which requires IT expertise [27]. The source code is publicly available on Bitbucket and was last updated in 2024 [28].

#### OpenPseudonymiser

Developed by the University of Nottingham, the OpenPseudonymiser is a desktop application that has been available since 2011 and generates pseudonyms using a hash function with a salt. It allows users to generate hashes for one or more columns in a CSV file. Since it is a standalone desktop application that processes files, no system administration knowledge is required, making it suitable for non-technical users. While the user manual indicates a source code revision in 2020, access to the software requires registration on the OpenPseudonymiser website [29].

#### ORCHESTRA Pseudonymization Tool

The ORCHESTRA Pseudonymization Tool (OPT), released in 2024, is implemented based on widely available office suites to support rapid deployment [30]. It supports namespaces and the management and pseudonymization of patient or proband identities as well as biosamples. Since it integrates with commonly used office tools, it is particularly user-friendly and does not require additional IT infrastructure or system administration skills. The code, updated in 2024, is publicly available on GitHub [31].

#### PID-Generator

Initially developed in 2000, the PID-Generator uses a deterministic, rule-based algorithm to generate pseudonyms and store them in a database [32]. It provides a command line interface (CLI) and requires manual setup and some configuration for rule-based pseudonym generation [33]. Deployment therefore requires advanced technical expertise. The tool is available for download on the project homepage, along with documentation in German, with no further development documented since its initial release [32].

#### Pseudonymization Service

The Pseudonymization Service was first launched in 2004, extending the PID-Generator with a symmetric-key algorithm to generate pseudonyms of fixed length [23]. Originally using a CLI, later updates introduced a desktop GUI, enabling configuration and data processing through specific configuration files and physical media. Initial setup is done via configuration files, which may require some technical expertise. The documentation available on the project homepage is outdated and only covers the first version of the software in German [34]. Access to the service, which was last updated in 2019, must be requested from the developer [35].

#### SPIDER

Launched in 2022, the software Secure Privacy-preserving Identity management in Distributed Environments for Research (SPIDER) is provided via the European Platform on Rare Disease Registration (EU RD Platform). Unlike EUPID, SPIDER is exclusively intended for research on rare diseases [36, 37]. SPIDER is provided as SaaS solution and also offers users the ability to access it via the web-based interface on the ERDRI platform, allowing the tool to be used directly in browsers. Alternatively, it is possible to develop a dedicated SPIDER client as a stand-alone or plug-in solution, which may require programming knowledge.

The source code is not publicly accessible, so potential updates cannot be verified. However, the EU RD Platform website provides comprehensive documentation and detailed training videos [38].

### Comparison

When comparing the identifying tools, it can first be seen that they have been implemented for distinct application scenarios:

1. Software-as–a-Service solutions: EUPID and SPIDER are provided as hosted-services that can either be used within EU platforms or integrated into other applications. This means that specific legal bases (e.g., consent) and contracts are needed to use them in a compliant manner. Both solutions offer comparable functionalities (pseudonym spaces, data linkage, batch processing, and an API), with SPIDER focusing on rare disease research.
2. Pseudonymization of existing data: The primary focus of CRATE is on pseudonymizing existing databases with structured, and potentially unstructured, data for further research use. CRATE supports pseudonym spaces, dataset linkage, and batch processing, but does not provide an API.
3. Pseudonymization of existing as well as newly collected data: ALIIAS, gPAS, Mainzelliste, OpenPseudonymiser, OPT, PID-Generator and the Pseudonymization Service all provide functionalities to pseudonymize new records in a transactional manner, making them suited for integration into data collection processes, for example.

The remainder of this comparison focuses on the largest group of tools, i.e., those designed to support the pseudonymization of existing as well as newly collected data.

Multi-center studies use data from multiple institutions, which often requires tools to support non-overlapping pseudonym spaces. Considering that study participants may switch between different centers, record linkage becomes necessary to maintain accurate tracking of participant data across centers. To facilitate the data and workflow integration between the participating institutions, there are often requirements to integrate their systems, e.g., requiring a central service with an API. Similarly, a central tool usually requires a web-based interface. In contrast, in single-center settings, tools are not always used by a larger group of individuals, so native user interfaces can be sufficient and API integration may not be necessary.

In short-term projects, where the focus is often on quick implementation and immediate results, standalone solutions with native interfaces and limited API integration are often sufficient. These solutions are typically easier to set up and maintain. In contrast, long-term projects often require more flexibility and support for a larger user group, making web-based services more suitable. The availability of an API can enable the integration with other systems, which is a typical requirement for long-term structures. Moreover, the ability to link and update the data managed by the service is likely to become critical.

Studies or projects dealing with small data often involve manual data collection, e.g., following a pre-defined study protocol, which may reduce the necessity for batch processing. Furthermore, projects managing only small amounts of data are also often short-term, single center projects that can be supported well with simpler tools offering only native interfaces and limited API integration. In contrast, projects involving big data usually require batch processing capabilities, which is often easier to achieve with a web-based service offering an API. Similarly, studies or projects that require integration with other systems and structures, e.g., for data collection or biobanking, are also easier to set up using an API, while more isolated standalone projects can often be carried out with simpler tools that don’t offer one.

**Fig. 3:**
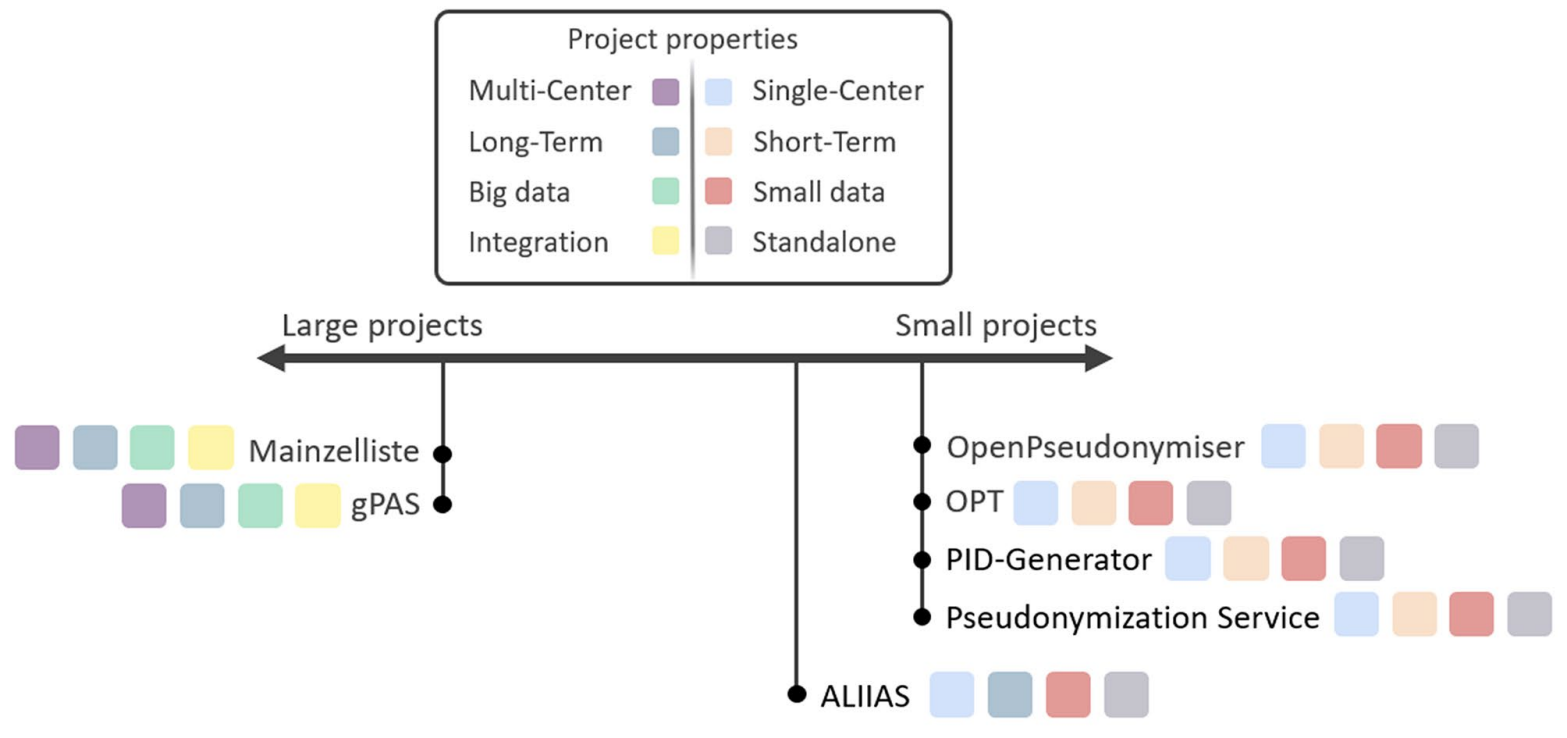
Suitability of pseudonymization tools for projects with different properties

Figure 3 illustrates the suitability of pseudonymization tools for projects with different properties. The comparison shows two extreme cases based on the example scenarios defined in the introduction: (A) large projects (multi-center, long-term, big data, integration) and (B) small projects (single-center, short-term, small data, standalone). The arrow illustrates the range between these two extremes. Tools such as Mainzelliste and gPAS are more suitable for complex projects, while OpenPseudonymiser, OPT, PID-Generator and the Pseudonymization Service are generally more suitable for smaller projects. ALIIAS could also be used for long-term projects, but is generally more suitable for small projects. The color coding indicates which project properties are tool addresses particularly well.

Most of the mentioned tools support both pseudonym spaces and record linkage. However, ALIIAS supports pseudonym spaces but not record linkage, making it more suitable for specific use cases, such as surveys. The Pseudonymization Service neither supports pseudonym spaces nor record linkage. Tools that support operations like deleting, updating or anonymizing pseudonymized data e.g., to correct an entry or to meet legal requirements (such as requests under GDPR data subject rights) include gPAS, the Mainzelliste and the OPT. Batch processing is supported by most tools, with the exception of ALIIAS, which only allows direct data entry. The OpenPseudonymiser and the Pseudonymization Service only support batch processing via prepared lists, such as CSV files, while direct entry is not possible. User interfaces vary between web-based and native applications. For long-term use, having a tool provided as a web-based service instead a locally installed software is often preferable, as it facilitates ongoing maintenance and adaptation to changing requirements. Web-based tools, including ALIIAS, gPAS, and Mainzelliste, allow flexible access via a browser. In contrast, native applications such as OpenPseudonymiser, OPT, PID-Generator, and the Pseudonymization Service require local installation. However, the OpenPseudonymiser and the OPT can be deployed quickly due to their minimal infrastructure requirements. Mainzelliste offers a modern, user-friendly GUI with a RESTful API, facilitating seamless integration with other systems. Similarly, gPAS supports integration but relies on a SOAP-based API instead of REST. Native tools like OpenPseudonymiser, OPT, PID-Generator, and the Pseudonymization Service offer GUIs but lack APIs. This makes them more suitable for deployments that do not require external programming integration.

## Discussion

### Principal findings and related work

Our review identified and we systematically analyzed ten pseudonymization tools for biomedical research and highlighted seven tools that demonstrate particular strengths in addressing key requirements of medical research projects. SPIDER and EUPID are options, if external SaaS offerings are needed and can be used. CRATE, on the other hand, is a tool specifically focusing on the pseudonymization of existing databases. For local deployments, two tools are particularly suited for large and two for smaller projects. In the context of larger projects (1) gPAS is well suited for multi-center studies as well as local long-term projects and it can also be combined with further tools for consent management and patient registration, and (2) the Mainzelliste, with its RESTful interface, supports both efficient data pseudonymization and flexible integration into existing research networks.

For short-term studies and smaller local projects, the (3) OpenPseudonymiser and the (4) OPT can be recommended, as they support the most features, including pseudonym spaces, record linkage and secondary pseudonymization.

There are also tools and services designed to handle data in different interoperability formats, such as the FHIR Pseudonymizer for clinical data [39].

Several articles and studies have previously addressed the topic of pseudonymization and its role in medical research. Kohlmayer et al. [10] explored the challenges of pseudonymization in the context of real-world data collection, emphasizing the importance of balancing data protection with research needs. Similarly, Lautenschläger et al. [12] provided a solution for the web-based management of pseudonymized data, focusing on scalability and security in distributed research environments. The European Union Agency for Cybersecurity (ENISA) outlines different pseudonymization scenarios and provides detailed technical recommendations on methods and best practices in its report [8]. However, it does not offer a comparison or even a recommendation of specific pseudonymization tools. Recent work by Gehrmann et al. [3] focused on the barriers to secondary use of medical data across research sites, pointing out that legal and technical challenges remain significant. It referred, for example, to the challenges arising from the use of different identifiers at different sites. These works primarily form the basis for understanding the complexity of selecting and implementing pseudonymization tools.

### Limitations and future work

This review focuses specifically on pseudonymization tools for medical re-search, which introduces several limitations.

First, our analysis does not include advanced privacy-enhancing techniques, which have gained traction in the field of biomedical and healthcare research, such as differential privacy [40]. However, while these techniques offer rigorous privacy guarantees, they operate on a different conceptual level and were outside the scope of our study. Pseudonymization serves a fundamentally different purpose, as it enables the collection and integration of patient data while implementing privacy-by-design principles, particularly under frameworks such as the GDPR, ensuring data protection even when full anonymization is not possible or needed.

Second, our study relies exclusively on literature- and documentation-based assessments without practical experimentation or benchmarking. This approach limits the ability to evaluate factors such as the user-friendliness or real-world performance. In future work, we plan to conduct empirical evaluations using representative medical datasets and application scenarios to provide a more practical comparison.

Finally, our search strategy primarily identified tools from European, particularly German, contexts. While this may indicate a selection bias, we would also like to emphasize that pseudonymization has gained significant prominence in the EU with the adoption of the GDPR, where it plays a major role, and that Germany has a long-established tradition of using pseudonymization in medical research.

## Conclusion

To the best of our knowledge, we presented the first systematic review of pseudonymization tools for biomedical research. Our work shows that pseudonymization tools can be compared along different dimensions, which can in turn be used to identify those that are specifically well suited for supporting certain types of medical research projects.

## Electronic supplementary material

Below is the link to the electronic supplementary material.

Supplementary Material 1

Supplementary Material 2

Supplementary Material 3

## Data Availability

All relevant data and materials are included in this article and its appendix.

## Abbreviations

AI: Artificial Intelligence
API: Application Programming Interface
CLI: Command-Line Interface
CSV: Comma Separated Value
EC-JRC: European Commission – Joint Research Centre
ENISA: European Union Agency for Cybersecurity
ERDRI: European Rare Disease Registry Infrastructure
EU: European Union
EU RD Platform: European Platform on Rare Disease Registration
EUPID: European Unified Patient Identity Management
GDPR: General Data Protection Regulation
gPAS: Generic Pseudonym Administration Service
GUI: Graphical User Interface
NLP: Natural Language Processing
PID: Personal Identifiers
PRISMA: Preferred Reporting Items for Systematic Reviews and Meta-Analyses
REST: Representational State Transfer
SaaS: Software as a Service
SOAP: Simple Object Access Protocol
SPIDER: Secure Privacy-preserving Identity management in Distributed Environments for Research
TMF: Technologies, Methods and Infrastructure for Networked Medical Research e.V
