# Supplementary material for: Pseudonymization tools for medical research: a systematic review
Source: BMC Med Inform Decis Mak. 2025 Mar 12;25:128. doi: 10.1186/s12911-025-02958-0 (PMC11905493; doi:10.1186/s12911-025-02958-0)
Supplement: Supplementary file 1 — Supplementary Material 1 [file 12911_2025_2958_MOESM1_ESM.docx]

| PubMed | ("pseudonyms" OR "pseudonym" OR "pseudo-anonymous" OR "pseudo-anonymization" OR "pseudo-anonymisation" OR "linked-anonymous" OR "linked-anonymization" OR "linked-anonymisation" OR "pseudonymity" OR "pseudonymization" OR "pseudonymisation" OR "pseudonymized" OR "pseudonymised" OR "pseudonymous") | AND |
| --- | --- | --- |
|  | ("Tool" OR "Software" OR "Service" OR "Application") |  |
|  | [tiab] | IN |

| Web of Science  (*Core Collection*) | (pseudonyms OR pseudonym OR pseudo-anonymous OR pseudo-anonymization OR pseudo-anonymisation OR linked-anonymous OR linked-anonymization OR linked-anonymisation OR pseudonymity OR pseudonymization OR pseudonymisation OR pseudonymized OR pseudonymised OR pseudonymous) | AND |
| --- | --- | --- |
|  | (Tool OR Software OR Service OR Application) |  |
|  | [Topic] | IN |
